# Gestational diabetes alters subgingival pathobiont composition

**DOI:** 10.2340/aos.v85.45789

**Published:** 2026-04-15

**Authors:** Fatih Cömert, Funda Yalçın, Nursen Topçuoğlu, Oya Kaya-Şimşek, Oya Demirci, Silvi Domnori, Ulku Baser

**Affiliations:** a Department of Periodontology, Faculty of Dentistry, Istanbul University, Istanbul, Türkiye; b Department of Oral Microbiology, Faculty of Dentistry, Istanbul University, Istanbul, Türkiye; c Department of Perinatology, Zeynep Kamil Women and Children’s Diseases Training and Research Hospital, Health Science University, Istanbul, Türkiye; d Department of Periodontology, Faculty of Dentistry, Institute of Graduate Studies in Health Sciences, Istanbul University, Istanbul, Türkiye

**Keywords:** pregnancy, gestational diabetes mellitus, subgingival pathobionts, periodontal inflammation

## Abstract

**Objective:**

This cross-sectional study assesses the relationship between gestational diabetes mellitus (GDM) and periodontal dysbiosis by evaluating specific periodontal pathobionts (*Porphyromonas gingivalis, Prevotella intermedia, Tannerella forsythia*, and *Treponema denticola)*, clinical periodontal parameters, and periodontal inflamed surface area (PISA).

**Materials and methods:**

101 GDM and 98 non-GDM women of 16–36 weeks gestation were included. Clinical periodontal parameters were measured, and PISA values calculated. Subgingival plaque samples, collected from the deepest pockets, were analyzed, to assess the amount of periodontopathogens. Associations were assessed using binary logistic regression and path analysis.

**Results:**

The GDM group showed higher clinical parameters and PISA values (*p* < 0.001). While *P. gingivalis* levels were similar in both groups (*p* = 0.924) and unrelated to the presence of GDM in path analysis (*p* = 0.055), *P. intermedia* and *T. denticola* levels were found higher in the non-GDM group (*p* < 0.05, *p* < 0.001) and negatively associated with the presence of GDM (*p* < 0.001, *p* = 0.002). *P. intermedia* increased with gestation week (*p* = 0.044). Elevated *T. forsythia* levels were observed to increase GDM risk 1.208-fold in regression analysis (*p* = 0.002).

**Conclusion:**

Increasing sex hormone levels accompanied by the presence of GDM may alter subgingival pathobiont composition. In patients with GDM, there is a heightened burden of *T. forsythia* in subgingival zones, where *P. intermedia* finds less favorable conditions.

## Introduction

The etiopathogenesis of periodontal diseases is described by the polymicrobial synergy and dysbiosis model of Hajishengallis & Lamont [1]. This model suggests that pathobionts and certain keystone pathogens proliferate in proportion to symbiotic bacteria. The immune subversion mechanisms that these microorganisms possess allow them to induce an inflammatory response, which, despite its activation, fails to translate into an effective immune response. Consequently, inflammophilic bacteria capitalize on the inflammatory environment, intensifying the inflammation and accelerating periodontal destruction [2].

The transition into a diseased condition is significantly influenced by immunopathogenesis, which, is itself shaped by risk factors, including genetic predisposition, environmental factors, and systemic conditions [3].

As a metabolic disease characterized by systemic inflammation, diabetes has long been studied in conjunction with periodontitis, to explore their potentially common inflammatory etiopathogenesis. The interplay between diabetes and periodontal inflammation is well-documented, with overt diabetes exacerbating periodontitis and vice versa, that is, periodontitis potentially worsening glycemic control [3–5].

Gestational diabetes mellitus (GDM) is defined as glucose intolerance with onset diagnosis during pregnancy. It is characterized by insulin resistance stemming from the effect of sex steroid hormones [6]. The blocking effect these hormones may exert on insulin is described as a contra-insulin effect, usually observed at about 20–24 weeks of pregnancy [7]. Inflammation represents an important and frequent contributor to complications of pregnancy [8]. Globally, GDM represents the majority (84%) of hyperglycemia cases in pregnancy, as stated by the International Diabetes Federation (IDF), which estimates that one in six live births are affected by maternal hyperglycemia [9].

Both pregnancy and diabetes impact periodontal health independently. GDM, being a pregnancy-specific form of diabetes, is a global problem affecting both maternal and infant health. Its interaction with periodontal disease may influence maternal and infant health locally and systemically, by altering the periodontal microbiota.

During pregnancy, there is a marked rise in estrogen and progesterone levels [10]. Fluctuations in these hormone levels, specifically increasing until the 8^th^ month of pregnancy, can lead to higher vascular permeability [11]. This change often manifests within the oral cavity as pregnancy gingivitis, with increases in probing pocket depth (PPD) and bleeding on probing (BoP) [12, 13]. The resulting inflammatory condition fosters the presence of bacteria that thrive in a periodontally-compromised oral environment, which is also exacerbated by glycemic dysregulation and altered immune response.

Several bacterial species have been shown to increase during pregnancy. *Porphyromonas gingivalis* stands out as a keystone pathogen [1] with potent virulence factors such as gingipains and endotoxins promoting periodontal damage even at low abundance. It has been associated with worsening gingival inflammation during pregnancy [14] as well as pregnancy complications [15–17]. Bacteria such as *Prevotella intermedia* and *Prevotella melaninogenica* can utilize estradiol and progesterone as substitutes for vitamin K, promoting their proliferation in the subgingival environment [11, 18]. Studies have reported a peak in *P. intermedia* levels during the second trimester, which coincides with heightened gingival inflammation and subsides postpartum [11, 19–22]. These shifts in the subgingival microbiota also include other pathogenic species, such as *Tannerella forsythia* and *Treponema denticola*, both members of the red complex detected in the subgingival microbiota during pregnancy, with their virulence factors contributing to epithelial invasion, immune evasion, and enhanced tissue breakdown [15, 23]. Accumulated evidence implicates the role of *P. gingivalis, T. forsythia, T. denticola*, and *P. intermedia*, in pregnancy complications, including preterm delivery [15–17, 24–27]. These four bacterial species were selected for analysis based on their well-established role in periodontal disease progression and their reported associations with pregnancy complications. A recent systematic review [28] has highlighted the underexplored biological mechanisms connecting periodontal disease and GDM; hence, investigating the abundance of these bacteria in the context of GDM could provide a targeted frame to reflect potential microbial and clinical patterns.

The null hypothesis of our study states that GDM patients have the same periodontal microbiota as a pregnant woman without GDM. In addition to investigating the relationship between periodontopathogens and GDM, we calculated the periodontally inflamed surface area (PISA) to quantify the inflammatory condition in both patient groups. The aim of this study is to evaluate the subgingival pathobiont composition of pregnant women with and without gestational diabetes and investigate its associations with clinical parameters and PISA.

## Materials and methods

### Study design and population

**Figure 1:**
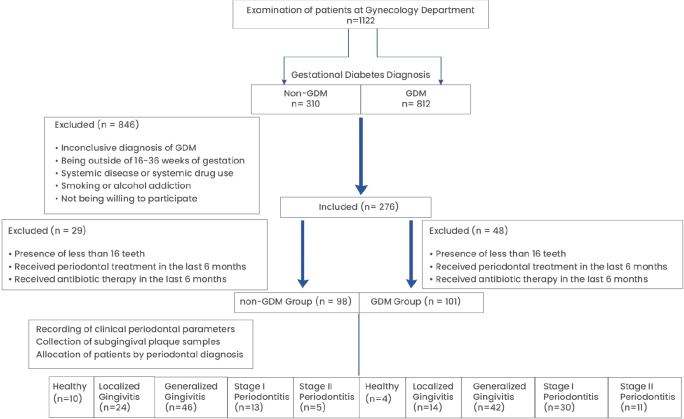
Flowchart of the study.

This cross-sectional study was conducted between March 2019 and June 2021 at two main research hospitals in Istanbul, Turkiye: (1) the Division of Endocrinology and Metabolism, Department of Internal Medicine, Faculty of Medicine, Istanbul University; and (2) the Department of Obstetrics and Gynecology, Zeynep Kamil Gynecology and Paediatrics and Research Hospital. The study was conducted and reported in accordance with the STROBE guidelines for observational studies [29]. Out of 1122 patients examined, 101 GDM and 98 non-GDM patients were included (Figure 1).

Inclusion criteria:

1. Gestational age of 16–36 weeks.
2. Diagnosed with or without GDM and no other systemic diseases.
3. Have more than 16 teeth excluding third molars.

Exclusion criteria:

1. History of cardiovascular, cerebrovascular, peripheral arterial, respiratory diseases, mental disorders (e.g. depression), rheumatoid arthritis, and osteoporosis.
2. Smoking or alcohol addiction.
3. Use of antibiotics within 6 weeks prior to sampling.
4. Use of medication affecting periodontal tissues.

This study was conducted in accordance with the Declaration of Helsinki as per the 2013 revision and approved by the Clinical Research Ethics Committee of Istanbul University Faculty of Dentistry (Approval No: 2019/22). Informed consent was obtained from all patients prior to their inclusion in the study.

### Diagnosis of GDM

GDM was diagnosed according to the American Diabetes Association criteria (2013), that is, a 75 g oral glucose tolerance test was administered at 16–36 weeks of gestation, with fasting plasma glucose measured at 1 and 2 h. Threshold values for GDM diagnosis were fasting ≥ 92 mg/dl (5.1 mmol/l); 1 h ≥ 180 mg/dl (10.0 mmol/l); and 2 h ≥ 153 mg/dl (8.5 mml/l).

### Clinical examination

Demographic information, medical and dental anamnesis of the participants were recorded via a questionnaire.

Clinical periodontal parameters including gingival index (GI) [12], plaque index (PI) [30], PPD, clinical attachment level (CAL), and BoP [31] were recorded. Oral examination was performed using a Williams^*^ type periodontal probe, recording measurements at six sites per tooth (mesiobuccal, midbuccal, distobuccal, mesiolingual, midlingual and distolingual). All measurements were performed by a single investigator (F.C.).

To quantify the amount of inflamed periodontal tissue and as such, the inflammatory burden, we calculated PISA values, using the formula by Nesse et al. [32], based on CAL/PPD, gingival recession and BoP data. PISA is expressed in square millimeters (mm^2^), representing the total surface area of the bleeding pocket epithelium.

Clinical diagnosis was done according to the 2017 Classification of Periodontal and Peri-Implant Diseases and Conditions [33]. The patients were categorized into five groups: Healthy, with Localized Gingivitis, with Generalized Gingivitis, with Periodontitis Stage I or with Periodontitis Stage II.

### Subgingival plaque sampling

Subgingival plaque samples were collected from each participant’s four teeth with the deepest pockets. Cotton pellets were placed in the buccal and lingual sulci to prevent salivary contamination. Supragingival plaque was carefully wiped from the tooth surfaces using a sterile cotton pellet, and then subgingival plaque samples were obtained using standard absorbent paper points (#40). A total of 16 samples were taken from 4 sites per tooth (mesiobuccal, distobuccal, mesiolingual/-palatal and distolingual/-palatal) where paper points were inserted and left in place for 30 s. Samples contaminated with blood during collection were excluded. The collected paper points were placed in two Eppendorf tubes of eight samples each. Samples were initially stored in a refrigerator at +4°C, then transferred to −80°C ultra-low temperature freezers (New Brunswick Scientific, USA) for long-term storage.

### DNA extraction procedure from plaque sample

Genomic DNA was extracted from paper points collected from patients using E.Z.N.A Bacterial DNA Kit (Omega Bio-tek^†^). Initially, 100 µl of TE buffer was added to the paper points which were then vortexed thoroughly, and subsequently, manufacturer’s protocol was followed as indicated. Finally, DNA was eluted in 60 µl of elution buffer. DNA concentration and purity were assessed using a NanoDrop spectrophotometer (Thermo Fisher Scientific^‡^), measuring absorbance at 230, 260, and 280 nm and calculating 260/280 and 260/230 ratios. Isolated DNA samples were stored at −20°C until further analysis.

### Quantitative PCR analysis

Species-specific quantitative PCR (qPCR) method was used to determine the relative amounts of *P. gingivalis, P. intermedia, T. forsythia*, and *T. denticola* in the isolated DNA samples. The specific primers for each target bacteria are shown in Table 1.

**Table 1:** qPCR Primer sequences for each microorganism.

Microorganism	Primer sequences (5′→3′)	Amplicon length (bp)	References
*P. gingivalis*	F: AGGCAGCTTGCCATACTGCG R: ACTGTTAGCAACTACCGATGT	405	Bogen, and Slots, 1999
*P. intermedia*	F: GACCCGAACGCAAAATACAT R: AGGGCGAAAAGAACGTTAGG	130	Barbosa 2015
*T. denticola*	F: CCTTGAACAAAAACCGGAAA R: GGGAAAAGCAGGAAGCATAA	109	Hyvarinen et al., 2009
*T. forsythia*	F: GCGTATGTAACCTGCCCGCA R: TGCTTCAGTGTCCAGTTATACCT	641	Ashimato et al.,1996

Each 20 μL reaction mixture contained 10 μL of 2x GoTaq qPCR Master Mix (Promega), 0.5 μM of each primer, 2 μL of template DNA, and nuclease-free water. Amplification was conducted with an initial enzyme activation at 95°C for 2 min, followed by 40 cycles of 95°C for 15 s and 60°C for 1 min. A melting curve analysis (60–95°C) was performed to confirm amplification specificity. Two no-template controls were included in each plate to rule out possible contamination. All reactions were performed in a LightCycler480 Real-Time PCR System (Roche^§^).

Following qPCR, Ct values were obtained and used for relative quantification analysis. Comparative analyses between patient groups were performed by using fold changes calculated by Ct values. For visualization purposes, Ct values were normalized to the average Ct values (Ct_sample – Ct_average) of positive samples for each target microorganism. Group and sampling time comparisons were made using the fold-change rates obtained for each sample.

The primary outcome measure of this study is the quantification of *P. gingivalis*, *P. intermedia*, *T. forsythia*, and *T. denticola* in subgingival plaque samples using qPCR to compare bacterial loads between pregnant women with and without GDM. The secondary outcome measures include clinical periodontal parameters, PISA, and periodontal diagnosis classification to assess differences in periodontal health between the groups. The bacterial quantification values and all clinical periodontal parameters were treated as continuous variables, while sociodemographic features and periodontal diagnosis groups were categorized for statistical analysis.

### Statistical analysis

Our reference in estimating sample size were the studies by Gogeneni et al. [34] and Ganiger et al. [35]. The sample size was calculated using G*Power (version 3.1.9.2^¶^) with a two-sided independent samples t-test, Type 1 error of 5%, Type 2 error of 20% corresponding to 80% Power, and an effect size of 0.45, yielding 79 participants per group. To account for potential losses, 100 participants per group were planned, totaling to a sample size of 200.

Data were analyzed using IBM SPSS V23^‖^. Normality was assessed with the Kolmogorov-Smirnov Test. Due to violations of normality assumptions in several variables, non-parametric analyses were conducted. Mann Whitney U Test was used for group comparisons of continuous variables. Categorical data were analyzed using the Pearson Chi-Square Test, Yates’ correction, or Fisher’s Exact Test, as appropriate. Binary logistic regression analysis was used to examine the risk factors affecting GDM. Model fit was evaluated using the Hosmer-Lemeshow goodness-of-fit test. Bacterial count was compared across the periodontal condition subgroups using Mann Whitney U test. Path analysis was used to identify potential factors affecting bacterial count, where clinical periodontal parameters, systemic and behavioral factors were treated as predictors, based on biological plausibility and existing literature [11, 14, 15, 36, 37]. Results were presented as number (percentage) for categorical data and mean ± standard deviation and median (minimum–maximum) for quantitative data, with significance set at *P < 0.05* for all analyses.

## Results

Continuous variables were categorized for descriptive comparison. Age was grouped as 17–25, 26–35, and ≥ 36 years [38,39]. BMI was classified according to the WHO criteria as < 25 (normal), 25–29.9 (overweight), and ≥ 30 kg/m² (obese) [40]. Education level and income levels were categorized into low, middle, and high based on self-reported answers; numeric thresholds were not applied. Number of children and pregnancies were grouped into clinically relevant ranges (e.g. 0–1, 2, and ≥ 3). Brushing habits and interdental cleaning habits were categorized by frequency as none, irregular and twice or once a day, respectively. Statistically significant differences were found between the groups for age, BMI, number of children, and socio-economic level *(P < 0.001).* Number of pregnancies differed between the groups with GDM group participants mostly on their third pregnancy and above, while the non-GDM group participants were either in their first or second pregnancy (*P = 0.009*). Gestational week was found to be similar as 28.56 ± 3.6 in the non-GDM group and 28.95 ± 2.5 in the GDM group (*P = 0.866*). Brushing habits were found to be significantly different between the groups, while interdental cleaning was similar and very close to statistical significance (*P < 0.001 and P = 0.052*, respectively) (Table 2).

**Table 2:** Comparison of demographic data between the groups.

	Group		*p*
	Non-GDM (*n* = 98)	GDM (*n* = 101)	
**Age (year)**			
Median (Min–Max)	28 (17–44)	34 (20–43)	**< 0.001^b^**
Ages 17–25	27 (27.6)	11 (10.9)	**< 0.001^c^**
Ages 26–35	52 (53.1)	56 (55.4)	
Ages 36 and above	19 (19.4)	34 (33.7)	
**BMI *n* (%)**			
Median (Min–Max)	27.8 (18.7–44.9)	31.6 (19.5–46.7)	**< 0.001^b^**
Normal weight (BMI < 25)	28 (28.6)	8 (7.9)	**< 0.001^a^**
Overweight (BMI 25–29.9)	35 (35.7)	31 (30.7)	
Obesity (BMI ≥ 30)	35 (35.7)	62 (61.4)	
**Education level *n* (%)**			
Low (Literate/Elementary)	44 (44.9)	61 (60.4)	**0.03^c^**
Middle (Secondary education)	22 (22.4)	17 (16.8)	
High (University)	32 (32.7)	23 (22.8)	
**Income rate *n* (%)**			
Low	61 (62.2)A	76 (75.2)A	**0.026^c^**
Middle	23 (23.5)A	20 (19.8)A	
High	14 (14.3)A	5 (5)B	
**GDM history %**			
No GDM history	44 (97.8)A	49 (69)A	**< 0.001^c^**
GDM history present	1 (2.2)A	22 (31)B	
**Nr. of children *n* (%)**			
0–1 child	77 (78.6)	56 (55.4)	**< 0.001^c^**
2 children	17 (17.3)	31 (30.7)	
3 children and above	4 (4.1)	14 (13.9)	
**Brushing habits *n* (%)**			
No brushing	32 (32.7)A	9 (8.9)B	**< 0.001^a^**
2 times a day	34 (34.7)A	30 (29.7)A	
Irregular	32 (32.7)A	62 (61.4)B	
**Interdental cleaning *n* (%)**			
No cleaning	65 (66.3)	82 (81.2)	0.052
Irregular	26 (26.5)	16 (15.8)	
Once a day	7 (7.1)	3 (3)	

GDM: gestational diabetes mellitus; BMI: body mass index.

a Pearson Chi-Square Test;

b Mann Whitney U;

c Fisher’s Exact Test used for 2x2 comparisons or Somers’d/Gamma association for ordinal data, used where appropriate; A–B: There is no difference between groups with the same letter; Significance set at *p* < 0.05.

**Figure 2:**
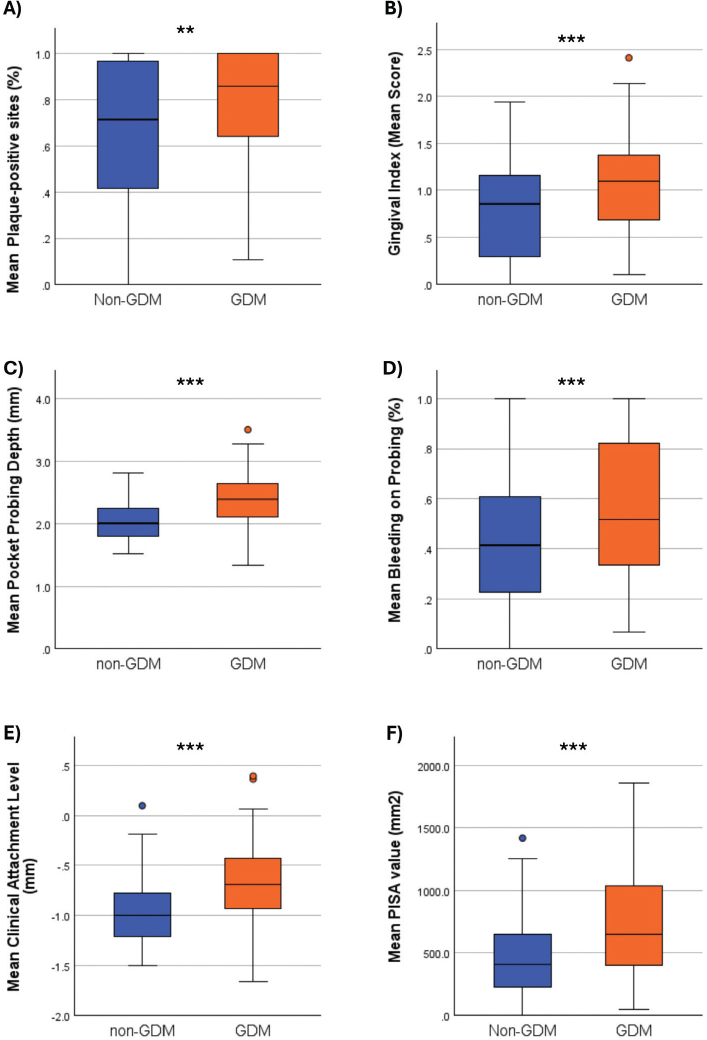
Comparison of periodontal parameters (A) PI: Plaque Index; (B) GI: Gingival Index, (C) PPD: Periodontal Pocket Depth; (D) BoP: Bleeding on Probing; (E) CAL: Clinical Attachment Loss; (F) PISA: Periodontal inflamed surface area. Mann Whitney U Test. * *p* < 0.05. ** *p* < 0.01. *** *p* < 0.001.

Statistically significant differences were observed in PI, GI, PPD, BoP, and CAL values between the groups, with all parameters higher in the GDM group *(P = 0.006* for PI and *P ≤ 0.001* for the other parameters). PISA values were also significantly higher in the GDM group compared to the non-GDM group *(P < 0.001)* (Figure 2).

**Figure 3:**
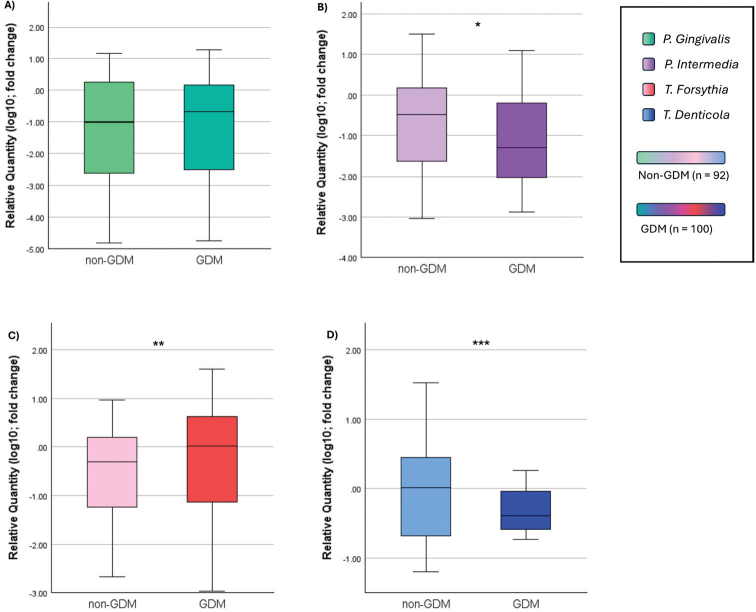
Periodontopathogen distribution without classification of periodontal disease. (A) *P. gingivalis – Porphyromonas gingivalis;* (B) *P. intermedia: Prevotella intermedia;* (C) *T. forsythia: Tanerella forsythia;* (D) *T. denticola – Treponema denticola.* Values were transformed using log(10) to normalize distributions for graphical visualization only; statistical analysis was conducted on raw data. Mann Whitney U test. * *p* < 0.05. ** *p* < 0.01. *** *p* < 0.001.

Of the 98 non-GDM patients, bacterial counts were evaluated in 92; among the 101 GDM patients, only one dataset was missing. The exact n values for each subgroup are indicated in Figure 3. No statistically significant difference was found for *P. gingivalis* prevalence between the GDM and non-GDM groups (*P = 0.924*). However, *P. intermedia* (*P = 0.044*), *T. forsythia (P = 0.002)* and *T. denticola (P < 0.001)* distributions differed significantly with *T. forsythia* more frequent in the GDM group, while *P. intermedia* and *T. denticola* were significantly more prevalent in the non-GDM group (Figure 3).

**Figure 4:**
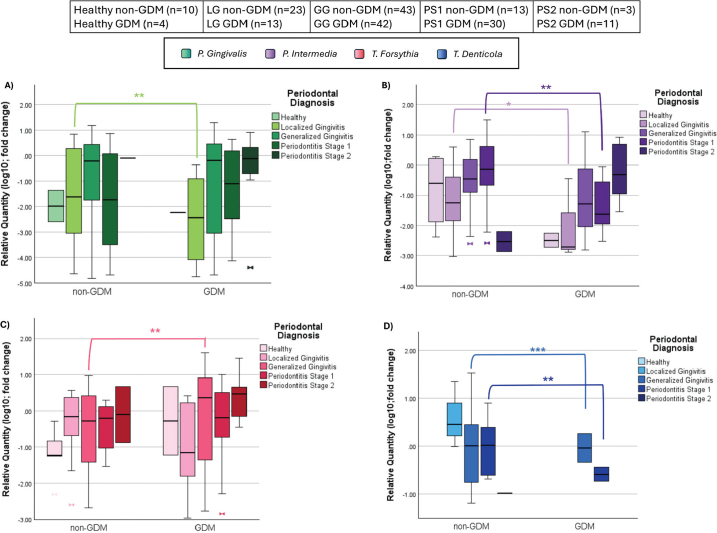
(A) Periodontopathogen distribution across diagnosis in *P. gingivalis*; (B) Periodontopathogen distribution across diagnosis in *P. intermedia*; (C) Periodontopathogen distribution across diagnosis in *T. forsythia*. (D) Periodontopathogen distribution across diagnosis in *T. denticola* (*LG – Localized Gingivitis, GG – Generalized Gingivitis, PS1 – Periodontitis Stage 1, PS2 – Periodontitis Stage 2);* non-GDM: pregnant without gestational diabetes mellitus, GDM: pregnant with gestational diabetes mellitus, n: number). Values were transformed using log(10) to normalize distributions for graphical visualization only; statistical analysis was conducted on raw data. Mann Whitney U Test. * *p* < 0.05. ** *p* < 0.01. *** *p* < 0.001.

No significant difference in bacterial levels was observed in healthy periodontium between GDM and non-GDM groups (Figure 4).

*P. gingivalis* and *P. intermedia* distributions differed significantly between 13 GDM and 23 non-GDM women diagnosed with localized gingivitis *(Figure 4A and B; P = 0.004)*, while *T. forsythia* and *T. denticola* showed no significant difference (Figure 4C and D).

No significant differences were found for *P. gingivalis* and *P. intermedia* levels between 42 GDM and 43 non-GDM women with generalized gingivitis. *T. forsythia* levels were higher in the GDM group *(median 1.93 vs. 0.14; P = 0.022)* while *T. denticola* levels were higher in the non-GDM group *(P = 0.001)* (Figure 4).

Among 30 GDM and 13 non-GDM women with stage I periodontitis, *P. intermedia* differed significantly (median 0 in GDM vs. 0.44 in non-GDM; *p* = 0.016) and *T. denticola* also showed a significant difference *(P = 0.008);* however, no significant differences were observed for *P. gingivalis (P = 0.642)* or *T. forsythia (P = 0.403)* (Figure 4).

There was no statistically significant difference between bacterial levels in 11 GDM and 3 non-GDM women with stage II periodontitis *(P = 0.301)* (Figure 4).

Regression analysis revealed that the risk of GDM increases with age *(1.114 times, P < 0.001)*, number of children *(1.746 times, P < 0.001)*, PI *(5.241 times, P = 0.002*), BoP *(7.284 times, P < 0.001)* and SCD *(11,898 times, P < 0.001).* Additionally, higher levels of *T. forsythia (1.208 times, P = 0.002)*, increased periodontal disease severity *(1.717 times, P = 0.001)* and PISA values *(1.002 times, P < 0.001)* were also associated with increasing GDM risk. Notably, the risk of GDM was 19,755 times higher in those with GDM history from their previous pregnancy *(P = 0.004).* Table 3 presents unadjusted odds ratios (ORs), 95% confidence intervals (CIs), and *p*-values for each independent variable.

**Table 3:** Risk factors affecting the relationship between gestational diabetes and periodontal condition by binary logistic regression analysis.

	Univariate OR (%95 CI)	*p* *p<0.05*
Age	1.114 (1.059–1.173)	**< 0.001**
BMI	1.001 (0.999–1.003)	0.513
Number of children	1.746 (1.301–2.341)	**< 0.001**
Gestation week	1.042 (0.951–1.142)	0.377
PI	5.241 (1.883–14.593)	**0.002**
BoP	7.284 (2.442–21.726)	**< 0.001**
PPD	11.898 (4.742–29.851)	**< 0.001**
*P. gingivalis*	0.979 (0.872–1.1)	0.724
*P. intermedia*	0.911 (0.782–1.063)	0.236
*T. forsythia*	1.208 (1.069–1.365)	**0.002**
*T. denticola*	0.245 (0.069–0.865)	**0.029**
Diagnosis	1.717 (1.266–2.328)	**0.001**
PISA	1.002 (1.001–1.003)	**< 0.001**
History of GDM in previous pregnancy (None)	Reference	
GDM in previous pregnancy	19.755 (2.556–152.681)	**0.004**
Education level (Low/Elementary)	Reference	
Intermediate (High school)	0.533 (0.252–1.128)	0.100
High (University)	0.496 (0.254–0.969)	**0.040**
Brushing habits (No brushing)	Reference	
2 times a day	3.137 (1.291–7.622)	**0.012**
Irregular	6.889 (2.933–16.178)	**< 0.001**
Interdental cleaning (No cleaning)	Reference	
Irregular	0.488 (0.242–0.985)	**0.045**
Once a day	0.34 (0.085–1.365)	0.128

BMI: body mass index; PI: plaque index; BoP: bleeding on probing; PPD: probing pocket depth; PISA: periodontal inflamed surface area; GDM: gestational diabetes mellitus; OR: odds ratio; CI: confidence internal.

**Figure 5:**
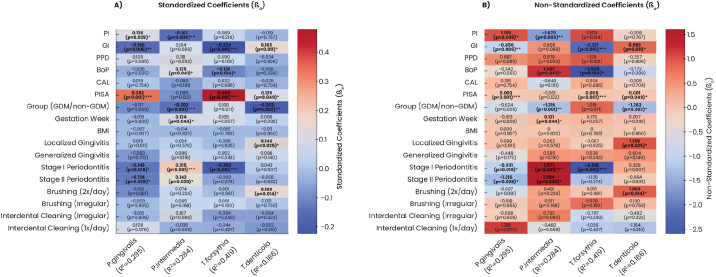
Heatmap of path analysis results with A. standardized and B. non-standardized coefficients. β_0_: Standardized Coefficients; β_1_: Non-Standardized Coefficients; *P. gingivalis: Porphyromonas gingivalis*; *P. intermedia: Prevotella intermedia*; *T. denticola: Treponema denticola*; *T. forsythia: Tannerella forsythia*; PI: Plaque Index; GI: Gingival Index; PPD: Periodontal Pocket Depth; BoP: Bleeding on Probing; CAL: Clinical Attachment Loss; PISA: Periodontally Inflamed Surface Area; non-GDM: pregnant without gestational diabetes mellitus; GDM: pregnant with gestational diabetes; BMI: Body Mass Index. * *p* < 0.05. ** *p* < 0.01. *** *p* < 0.001.

Significant path coefficients were identified for all the analyzed bacteria. For *P. gingivalis*, an increase in PI by one unit corresponded to a rise of 1.159 units (*p* = 0.039), while a unit increase in the GI led to a decrease of 0.856 units *(P = 0.006).* Similarly, *P. gingivalis* levels increased by 0.003 units with each unit increase in PISA value *(P < 0.001).* Compared to healthy individuals, levels of *P. gingivalis* were 0.931 units lower in stage I periodontitis *(P = 0.018)* and 1.316 units lower in stage II periodontitis *(P = 0.028)* (Figure 5).

For *P. intermedia*, a one-unit rise in PI resulted in a decrease of 1.67 units *(P = 0.009)*, while a unit increase in BoP increased its levels by 1.382 units *(P = 0.041).* The presence of GDM corresponded to 1.216 fewer units of *P. intermedia* compared to the non-GDM group *(P < 0.001).* Each additional week of gestation increased its levels by 0.121 units *(P = 0.044)* Those with stage I periodontitis showed an increase of 1.577 units *(P < 0.001)*, while stage II periodontitis was associated with a 1.584-unit increase *(P = 0.020)* as compared to periodontally healthy individuals (Figure 5).

For *T. forsythia*, a one-unit increase in GI and BoP led to decreases of 2.217 units *(P < 0.001)* and 2.539 units *(P = 0.014)*, respectively. In contrast, PISA value was positively associated with bacterium levels rising by 0.006 units per unit increase *(P < 0.001).* Compared to periodontally healthy individuals, *T. forsythia* levels were 2.516 units lower in stage I periodontitis participants (Figure 5).

For *T. denticola*, GI increases corresponded to a 0.933-unit rise *(P = 0.011)*, and PISA value increases led to a 0.001-unit rise *(P = 0.049).* In individuals with GDM, *T. denticola* levels were 1.263 units lower than non-GDM individuals *(P = 0.002).* Healthy individuals had 1.159 more units compared to those with localized gingivitis *(P = 0.025)*, while non-brushers had 1.063 more units than those brushing twice daily *(P = 0.014)* (Figure 5).

## Discussion

The surge of sex steroid hormones observed during pregnancy may be a cause for alterations in periodontal tissues and microbial profile responsible for periodontal disease. On the other hand, these hormones can lead to a condition known as insulin resistance, by reducing the effectiveness of insulin [7]. Our aim in this study was to evaluate how the systemic burden of these two different conditions manifests in the local oral tissues through periodontal pathobionts. Our findings indicate that periodontal microbiota and periodontal inflammation differ between patients with GDM and those without GDM.

According to our clinical parameters, periodontal inflammation was found higher in the GDM group. While *P. intermedia* and *T. denticola* were found higher in the non-GDM group, *T. forsythia* was found higher in the GDM group. *P. gingivalis* was found to be associated with the severity of periodontal disease rather than the presence or absence of GDM. Its levels in the subgingival plaque samples showed a statistically significant relationship with PISA values *(P < 0.001)* and it was more prevalent in patients with stage I and II periodontitis. Overall, the higher inflammatory burden in the GDM group with larger inflamed sulcular surface and increased bleeding [41] may shift the subgingival niche by increasing the availability of host-derived substrates within a low-redox environment. This shift may generate a nutrient-rich medium which can selectively favor these inflammophilic pathogens [42].

Individuals with GDM often present with older age, higher BMI, greater parity, a history of GDM in previous pregnancies, and less favorable sociodemographic conditions [43]. These characteristics were also observed in our study, consistent with existing literature [44]. Many studies have identified age as a risk factor for GDM development [34, 35, 45]. In addition to age, the difference we observed in periodontal condition could also be attributed to behavioral factors. However, due to the systemic burden of gestational diabetes, the distribution of microbiota still shows some differences regardless of these risk factors.

To our knowledge, the existing literature specifically investigating the presence of periodontopathogens in oral microflora in relation to gestational diabetes includes the studies by Gogeneni et al. [34], Ganiger et al. [35] and Dasanayake et al. [43].

Gogeneni et al. reported GDM to be associated with heightened infection by *P. gingivalis, Filifactor alocis*, and *T. denticola*, where the latter was only found statistically higher in GDM patients with gingivitis and an increase in systemic C-Reactive Protein levels, which contrasts our findings. However, Gogeneni et al. performed a conventional PCR analysis with binary results to evaluate the bacterial presence in saliva samples, which is not a specific reflection of periodontal microbial flora [34].

Ganiger et al. [35] found *P. gingivalis* and *P. intermedia* to be higher in GDM patients. Conversely, Dasanayake et al. [43] found no difference in *P. gingivalis* levels between GDM, and healthy pregnant women based on dental plaque and vaginal samples which is parallel to our results.

*P. intermedia*, the main pathogen responsible for pregnancy gingivitis [18–21], thrives in a progesterone-rich environment, linking it to pregnancy-related periodontal disease, as also demonstrated by previous studies [46, 47]. In our study, *P. intermedia* levels were significantly higher in the non-GDM group *(P < 0.001). P. intermedia* was detected in increasing numbers as the gestation week progressed. This trend coincides with the hormonal surge going into the third trimester, supporting the relationship between *P. intermedia* and pregnancy hormonal changes. Its relationship with BoP could be attributed to vascular changes at the bottom level of gingival pocket depth due to the effect of sex hormone surge during pregnancy [20].

*T. forsythia*, one of the main culprits of periodontal disease and tissue destruction, is frequently isolated from areas where the disease is actively progressing [23]. In our study, *T. forsythia* levels were significantly higher in areas with elevated GI and PISA value, which supports the thesis that it is responsible for active disease. *T. forsythia* levels were also significantly higher in GDM individuals (*P* = 0.002), suggesting its active involvement in periodontal disease and its modulation by GDM factors. Dasanayake et al. [43] reported higher *T. forsythia* levels in the vaginal fluids of GDM patients but found no difference in serum IgG or plaque samples. Similarly, Akherati et al. [48] observed higher prevalence of *T. forsythia* in the subgingival plaque of Type 2 diabetes mellitus patients with moderate periodontitis compared to non-diabetic patients with moderate periodontitis. Our binary logistic regression analysis indicated that the risk of GDM increased 1.208-fold with an increase in *T. forsythia* levels *(P = 0.002). T. forsythia* count was found to be associated with both increased PISA value and GDM. Capillary density in gingival tissues of patients with GDM was found to be higher compared to healthy non-pregnant controls, suggesting a cumulative effect of pregnancy and GDM in gingival microcirculation [41]. In this aspect, the additional systemic burden of GDM and increased sulcular fluid [41, 49], as seen through the higher PISA and BoP in the GDM group of our study, may increase the influx of serum proteins and hemoglobin into the pocket. This influx may expand local heme/iron availability (including albumin-associated pools and hemoglobin-derived heme) creating a niche which may favor a heme-scavenging pathobiont such as *T. forsythia* [50].

*T. denticola*, a highly mobile, invasive, gram-negative anaerobe and red complex member, is frequently isolated from periodontitis sites and associated with pregnancy complications [16]. Although it was only detected in a limited group of participants in our study, *T. denticola* was observed in greater numbers in the non-GDM group. According to path analysis, its association with GI and PISA might indicate that *T. denticola* is associated with periodontal inflammation *(P = 0.011 and P = 0.049 respectively).*

The strengths of our study include the adequate sample size, use of species-specific qPCR, plaque collection from the deepest subgingival sites throughout the mouth, comprehensive periodontal assessments of six sites per tooth for all clinical parameters and the incorporation of PISA values as a quantification of the inflamed periodontal surface area. Our PISA results align with Chaparro et al. who also found significantly higher PISA values in GDM patients compared to healthy pregnant women [51]. While we identified significant associations, one limitation of our study is its cross-sectional design, which limits the ability to establish a causality. Additionally, our sample was not matched for age, which, since GDM individuals are inherently older, may be a potential confounder in the observed associations. Dietary habits were also not assessed in our study, which may limit the interpretation of potential drivers of the hyperglycemic state and its relationship with the oral microbial profiles.

In considering the generalizability of this study’s findings, it is important to note that participants were recruited from tertiary care hospitals in Istanbul; hence, the results may not fully represent the wider population of pregnant women in Turkey – especially those living in areas with limited access to healthcare. Additionally, due to the restrictive exclusion criteria, our population may not capture the full spectrum of medical and dental complexity potentially seen in everyday practice; however, this was necessary to reduce the confounding factors. Future studies in more diverse populations, incorporating dietary assessments are needed to confirm the broader applicability of these findings.

## Conclusion

In individuals with GDM, increasing sex hormone levels combined with dysregulated glucose metabolism, may contribute to alterations in subgingival pathobiont composition. There may be an interplay between this environment and elevated *T. forsythia* levels in this subgingival area, whereas this environment may create a niche that disfavors *P. intermedia*. Consequently, GDM is associated with a distinct periodontal microbial profile and increased inflammatory burden.

### Declaration of generative AI and AI-assisted technologies in the writing process

During the preparation of this manuscript, the authors only used ChatGPT to improve its linguistic quality through inquiry of wording suggestions. After using this tool, the authors reviewed and edited the content as needed and take full responsibility for the publication’s content.

## Data Availability

The data that support the findings of this study are available from the corresponding author upon reasonable request.

## Footnotes

* Hu-Friedy, Williams Probe 0.5 mm, REF PQW, USA.

† Omega Bio-tek, E.Z.N.A.® Bacterial DNA Kit, Cat. No. D3350-02, Norcross, GA, USA.

‡ Thermo Fisher Scientific, NanoDrop 2000 Spectrophotometer, Waltham, MA, USA.

§ Roche Diagnostics, LightCycler 480 Real-Time PCR System, Basel, Switzerland.

‖ IBM Corp., IBM SPSS Statistics for Windows, Version 23.0, Armonk, NY, USA.

¶ Heinrich Heine University, G*Power, Version 3.1.9.2, Düsseldorf, Germany.
